# Evaluation of pH-Sensitive Polymeric Micelles Using Citraconic Amide Bonds for the Co-Delivery of Paclitaxel, Etoposide, and Rapamycin

**DOI:** 10.3390/pharmaceutics15010154

**Published:** 2023-01-01

**Authors:** Min Jeong Jo, Hee Ji Shin, Moon Sup Yoon, Seo Yeon Kim, Chae Eun Jin, Chun-Woong Park, Jin-Seok Kim, Dae Hwan Shin

**Affiliations:** 1 College of Pharmacy, Chungbuk National University, Cheongju 28160, Republic of Korea; 2 Drug Information Research Institute (DIRI), College of Pharmacy, Sookmyung Women’s University, Cheongpa-ro 47-gil 100, Yongsan-gu, Seoul 04310, Republic of Korea

**Keywords:** gastric cancer, combination therapy, pH-sensitive polymeric micelles, combination index, pharmacokinetics

## Abstract

Paclitaxel (PTX), etoposide (ETP), and rapamycin (RAPA) have different mechanisms, allowing multiple pathways to be targeted simultaneously, effectively treating various cancers. However, these drugs have a low hydrosolubility, limiting clinical applications. Therefore, we used pH-sensitive polymeric micelles to effectively control the drug release in cancer cells and to improve the water solubility of PTX, ETP, and RAPA. The synergistic effect of PTX, ETP, and RAPA was evaluated in gastric cancer, and the combination index values were evaluated. Thin-film hydration was used to prepare PTX/ETP/RAPA-loaded mPEG-pH-PCL micelles, and various physicochemical properties of these micelles were evaluated. In vitro cytotoxicity, pH-sensitivity, drug release profiles, in vivo pharmacokinetics, and biodistribution studies of PTX/ETP/RAPA-loaded mPEG-pH-PCL micelles were evaluated. In the pH-sensitivity evaluation, the size of the micelles increased more rapidly at a pH of 5.5 than at a pH of 7.4. The release rate of each drug increased with decreasing pH values in PTX/ETP/RAPA-loaded mPEG-pH-PCL micelles. In vitro and in vivo studies demonstrated that PTX/ETP/RAPA-loaded mPEG-pH-PCL micelles exhibit different drug release behaviors depending on the pH of the tumor and normal tissues and increased bioavailability and circulation time in the blood than solutions. Therefore, we propose that PTX/ETP/RAPA- loaded mPEG-pH-PCL micelles are advantageous for gastric cancer treatment in drug delivery systems.

## 1. Introduction

Gastric cancer (GC), a solid malignancy that occurs within the gastric mucosa, is the third major reason for cancer-related mortality [1,2]. GC is often detected after it has already advanced or metastasized as it exhibits no symptoms in the early stages [3]. Surgery and chemotherapy have been used as the main treatment methods for GC; however, they do not sufficiently meet expectations due to inadequate accumulation between tumors, distant metastasis, drug resistance, and serious side effects [4,5,6,7]. Therefore, new therapeutic strategies for GC with improved efficacy and low toxicity need to be studied.

Drug combination therapy using several different mechanisms has emerged as a way to improve the limitations of multidrug resistance and side effects associated with high doses of single dosage forms [8,9]. Such combination therapy has been regarded as a promising therapeutic strategy as it reduces anticancer drug resistance, overcomes tumor heterogeneity, and provides synergistic anticancer effects [10,11].

Paclitaxel (PTX) is a representative taxane-based anticancer drug that has been widely used against various cancers, including ovarian cancer, lung cancer, GC, and breast cancer. PTX inhibits the proliferation of cancer cells by interfering with the separation of microtubules, which are used as a mechanism for division and self-replication during cell division [12,13]. Etoposide (ETP), a drug that can form a four-dimensional complex structure with DNA and topoisomerase II, causes damage by preventing DNA strand recombination. ETP is used to treat glioblastoma, lung cancer, sarcoma, non-lymphocytic leukemia, and lymphoma; however, it may result in myelosuppression as a side effect [14,15]. Rapamycin (RAPA) is well-known as a drug that affects cell signaling pathways that determine cell cycle progression and cell growth by selectively inhibiting the mammalian target of rapamycin (mTOR) [16,17]. Previous studies have shown that the two-drug combination of PTX, ETP, and RAPA has a synergistic effect in various carcinomas. Wang et al. demonstrated that the formulation co-loaded with PTX and ETP enhances cytotoxicity in MG63 and Saos-2 osteosarcoma cell lines more than when each of these drugs is used alone [18]. Shafer et al. demonstrated that RAPA enhances the effects of PTX by increasing tubulin polymerization and acetylation, inhibiting cell proliferation, and inducing apoptosis in endometrial cancer cells [19]. Itamochi et al. showed that chemotherapy in combination with RAPA and ETP produced a synergistic cytotoxic effect in ovarian cancer cells and extend the survival of mice with ovarian cancer xenografts [20]. In addition, it has been reported that combinations of drugs with similar mechanisms to PTX, ETP, and RAPA are effective in GC. Yildiz et al. demonstrated the moderate effectiveness and safety of the combination of oral ETP and docetaxel as a second-line treatment for advanced GC after failure of platinum-based therapy [21]. Fukamachi et al. found that the Wnt-mTOR pathway is strongly involved in the growth regulation of diffuse GC initiating cells, and based on this, they demonstrated that mTOR inhibitors and checkpoint inhibitors could be useful in treating a subset of diffuse GCs [22]. Zhang et al. demonstrated that BEZ235, a dual PI3K/mTOR inhibitor, enhances the effects of nab-paclitaxel in GC through regulating the PI3K/mTOR pathway and inhibiting cell proliferation [23]. Based on these results, we predict that the combination of PTX, ETP, and RAPA will be effective in GC cell lines. However, all of these drugs are poorly soluble in water, limiting their clinical application.

Polymeric micelles have been widely used as a method to overcome the low water solubility of hydrophobic drugs [24,25]. Polymeric micelles are known to have advantages such as excellent biocompatibility, extended blood circulation time after intravenous injection, and effective tumor targeting [26,27]. However, drug release after in vivo administration is generally uncontrolled, limiting the rate of drug accumulation in tumors [28,29]. To overcome these problems, pH-sensitive polymeric micelles for controlled drug release have emerged as an effective strategy [30,31,32]. Such pH-sensitive polymeric micelles can trigger drug release by using the pH differences between tumor tissues and normal tissues [33,34,35]. Previous studies have reported that the pH of tumors is slightly acidic (pH < 7.0), whereas that of normal tissues remains constant at a pH of 7.2–7.4 [36,37,38]. As a result, pH-sensitive polymeric micelles maintain a stable state in normal tissues, then reach the tumor site through the enhanced permeation and retention (EPR) effect and change to an unstable state in response to the low pH of the tumor and release the drugs [39,40,41].

Methoxy poly(ethylene glycol)-*b*-poly(ε-caprolactone; mPEG-*b*-PCL) is an amphiphilic block copolymer composed of a hydrophilic PEG outer shell and a hydrophobic PCL inner core [42]. Cao et al. reported that novel pH-sensitive mPEG-pH-PCL micelles were successfully synthesized by introducing weak acid cleavable citraconic acid amide linkages into the copolymer. Moreover, a previous study has shown that the hydroxyl end group of PEG is transferred to an amino group, and citraconic anhydride is used to link the PCL and PEG blocks [43]. Therefore, the drug-encapsulated pH-sensitive mPEG-PCL micelles release the drug by breaking the citraconic amide bond through the weak acidity of cancer cells.

**Figure 1:**
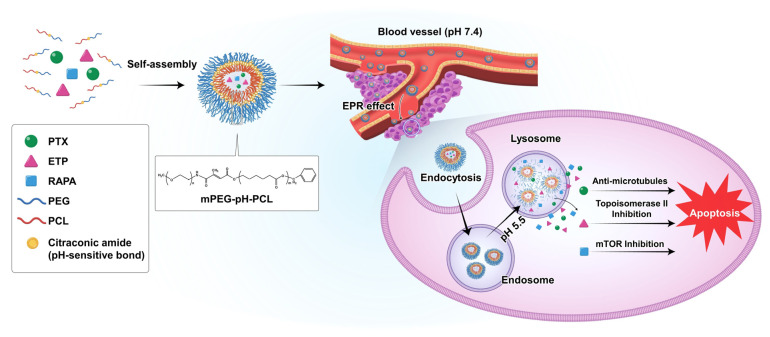
Schematic diagram of the drug release of paclitaxel (PTX), etoposide (ETP), and rapamycin (RAPA)-loaded mPEG-pH-PCL micelles in response to pH changes.

In this study, we used pH-sensitive mPEG-pH-PCL copolymer micelles as carriers for the controlled drug release and delivery of anti-cancer drugs (Figure 1). In addition, we assessed the combination index (CI) of PTX, ETP, and RAPA and investigated the in vitro cytotoxicity, in vitro pH-sensitivity assessment, in vitro release profiles, and pharmacokinetic profiles of micelles.

## 2. Materials and Methods

### 2.1. Materials and Reagents

Methoxy poly(ethylene glycol)-*b*-poly(ε-caprolactone) copolymer with citraconic amide as a pH-sensitive bond (mPEG-pH-PCL) was purchased from Creative PEGWorks (Chapel Hill, NC, USA). Cremophor EL^®^, dimethyl sulfoxide (DMSO), thiazolyl blue tetrazolium bromide (MTT), and crystal violet were purchased from Sigma-Aldrich Corp. (St. Louis, MO, USA). Distilled water (DW), ethanol (EtOH), acetonitrile (ACN), and methanol (MeOH) were purchased from Fisher Scientific (Waltham, MA, USA). PTX, RAPA, and sorafenib were purchased from LC Laboratories^®^ (Woburn, MA, USA). ETP was purchased from Tokyo Chemical Industry Co., Ltd. (Chuo-ku, Tokyo, Japan). Sodium chloride (NaCl), potassium phosphate monobasic (KH_2_PO_4_), potassium chloride (KCl), sodium phosphate dibasic anhydrous (Na_2_HPO_4_), and 0.1 N hydrochloric acid (HCI) standard solution were purchased from Duksan (Seoul, Korea) and were used for the manufacturing of phosphate-buffered saline (PBS). All other reagents and solvents were analytical grade or high-performance liquid chromatography (HPLC) grade.

### 2.2. Cell Line and Cell Culture

AGS-Luc2 cells, used as a human GC luciferase-expressing cell line, were obtained from the American Type Culture Collection (Manassas, VA, USA). Dulbecco’s phosphate-buffered saline (DPBS), Roswell Park Memorial Institute medium (RPMI 1640), fetal bovine serum (FBS), trypsin, and penicillin–streptomycin solution were purchased from Corning Inc. (Corning, NY, USA). AGS-Luc2 cells were cultured in RPMI 1640 medium using a CO_2_ incubator (Panasonic, Osaka, Japan) maintained at 37 °C and a 5% CO_2_ atmosphere. The media were supplemented with 1% (*w/v*) penicillin–streptomycin solution and 10% (*v*/*v*) FBS.

### 2.3. CI Analysis

CI analysis was conducted according to the Chou–Talalay method to assess the synergistic, antagonistic, or additive effects between the drugs [44]. The CI values were calculated using the following formula:

$$\mathrm{Combination}\;\mathrm{index}\;\left(\mathrm{CI}\right)=\frac{{\left(D\right)}_{1}}{{\left(D_{x}\right)}_{1}}+\frac{{\left(D\right)}_{2}}{{\left(D_{x}\right)}_{2}}+\frac{{\left(D\right)}_{3}}{{\left(D_{x}\right)}_{3}}$$

where (D_x_)_1_, (D_x_)_2_, and (D_x_)_3_ represent the half-maximal inhibitory concentration (IC_50_) values of a single drug, and (D)_1_, (D)_2_, and (D)_3_ represent the IC_50_ values of each drug when used in combination. CI < 1 indicates synergism, CI = 1 indicates additivity, and CI > 1 indicates antagonism.

### 2.4. Preparation of PTX/ETP/RAPA-Loaded Polymeric Micelles

The thin-film hydration method was used to prepare PTX/ETP/RAPA-loaded mPEG-pH-PCL micelles [45]. PTX (6 mg), ETP (6 mg), RAPA (3 mg), and mPEG-pH-PCL (150 mg) were dissolved in 1 mL of ACN. The organic solvent was evaporated under reduced pressure in a 60 °C water bath for 10 min using a rotary vacuum evaporator (EYELA^®^, Bohemia, NY, USA). Then, the thin film was hydrated with 1 mL of DW at 60 °C for 30 min. The PTX/ETP/RAPA-loaded micelle solution was centrifuged at 13,000 rpm for 5 min at 4 °C (Hanil Science Inc., Gimpo, Korea), and a 0.2 μm sterile filter was used to filter the supernatant (Corning, NY, USA).

### 2.5. Physicochemical Characterization of Micelles

A dynamic light-scattering (DLS) instrument was used to assess the particle size, zeta potential, and poly-dispersity index (PDI) of the PTX/ETP/RAPA-loaded mPEG-pH-PCL micelles (Litesizer 500, Anton Paar, Graz, Austria). The micelle solution was diluted 10-fold with DW before DLS measurement and with ACN before HPLC analysis. The drug loading (DL, %) and encapsulation efficiency (EE, %) of PTX, ETP, and RAPA loaded into micelles were calculated according to the following formulas [46,47]:

$$\mathrm{DL}\;\left(\%\right)=\frac{\mathrm{drug}\;\mathrm{weight}\;\mathrm{in}\;\mathrm{micelles}}{\mathrm{weight}\;\mathrm{of}\;\mathrm{the}\;\mathrm{micelles}}\times 100$$

$$\mathrm{EE}\;\left(\%\right)=\frac{\mathrm{drug}\;\mathrm{weight}\;\mathrm{in}\;\mathrm{micelles}}{\mathrm{weight}\;\mathrm{of}\;\mathrm{feeding}\;\mathrm{drug}}\times 100$$

### 2.6. Transmission Electron Microscopy (TEM) Observation

The morphology of PTX/ETP/RAPA-loaded mPEG-pH-PCL micelles was observed using TEM (JEOL Ltd., Tokyo, Japan). The micelle solution diluted with DW was dropped onto a 200-mesh copper grid, dried in a drying oven for 12 h at 60 °C, and measured at a voltage of 200 kV.

### 2.7. In Vitro pH-Sensitivity Assessment of Micelles

PTX/ETP/RAPA-loaded mPEG-pH-PCL micelles were diluted 10-fold with PBS (pH 7.4 and 5.5) to measure the particle size, zeta potential, and PDI. Each sample was stored at room temperature and measured at 0, 2, 4, 6, and 8 h. Particle size and PDI were measured to investigate the stability of the micelles in the tumor microenvironment (pH 5.5) and physiological conditions (pH 7.4).

### 2.8. In Vitro Cytotoxicity Assay

The cytotoxicity to AGS-Luc2 cells was evaluated by MTT assay [48]. AGS-Luc2 cells were seeded at a density of 5 × 10^3^ cells/well in 96-well plates. After 24 h of incubation, the medium was aspirated, and the cells were treated with free PTX, free ETP, free RAPA, free PTX/ETP/RAPA, PTX-loaded mPEG-pH-PCL micelles, ETP-loaded mPEG-pH-PCL micelles, RAPA-loaded mPEG-pH-PCL micelles, or PTX/ETP/RAPA-loaded mPEG-pH-PCL micelles. The drug-encapsulated mPEG-pH-PCL micelles were diluted 10-fold with RPMI medium. The free drugs were dissolved in 100 μL of DMSO and then diluted 1000-fold with RPMI medium to be used as an initial concentration. After incubation for 48 h, the medium was aspirated, and 100 μL of 0.5 mg/mL MTT solution was added. After 4 h of MTT treatment, the MTT solution was aspirated, and 100 μL of DMSO was added, followed by shaking at 200 rpm for 10 min using an orbital shaker (N-BIOTEK, NB-101S, Bucheon, Korea). The absorbance was measured using a microplate reader (Molecular Devices, Spectra Max ID3, San Jose, CA, USA) at a wavelength of 540 nm. All cell-related data processing was performed in GraphPad Prism v. 5 (GraphPad Software, La Jolla, CA, USA).

### 2.9. In Vitro Clonogenic Assay

Clonogenic assays were performed to assess the ability of single cells to form colonies and long-term cell survival [49,50]. AGS-Luc2 cells were seeded at a density of 200 cells/well in 6-well plates. After 24 h of incubation, the cells were treated with PTX-loaded mPEG-pH-PCL micelles, ETP-loaded mPEG-pH-PCL micelles, RAPA-loaded mPEG-pH-PCL micelles, or PTX/ETP/RAPA-loaded mPEG-pH-PCL micelles. The medium was removed after two weeks of incubation, and the colonies were stained with 1 mL of 0.5% w/v crystal violet. The dye was removed with fresh water after 30 min, and the number of colonies was counted. The following equation was used to calculate the colony formation [51]:

$$\mathrm{Colony}\;\mathrm{formation}\;\left(\%\right)=\frac{\mathrm{Number}\;\mathrm{of}\;\mathrm{colonies}\;\mathrm{in}\;\mathrm{the}\;\mathrm{treated}\;\mathrm{group}}{\mathrm{Number}\;\mathrm{of}\;\mathrm{colonies}\;\mathrm{in}\;\mathrm{the}\;\mathrm{control}\;\mathrm{group}}\times 100$$

### 2.10. In Vitro Drug Release Assay

The drug release of micelles encapsulated with PTX/ETP/RAPA was investigated using the dialysis method [52,53]. Briefly, PTX/ETP/RAPA-loaded mPEG-pH-PCL micelles were put in a dialysis membrane (MWCO = 20 kDa) and stirred in 2 L of PBS (pH 7.4 and 5.5) at 37 °C and 200 rpm. The pH of PBS (pH 7.4 and 5.5) was measured with a pH meter (Mettler Toledo, Columbus, OH, USA), and the pH values were titrated by 0.1 N HCI. At predetermined sampling times (0, 2, 4, 6, 8, 24, 48, 72, 168, 240, and 336 h), 20 µL of a sample was taken and diluted 10-fold with ACN. The concentrations of PTX, ETP, and RAPA were determined by HPLC. At 8, 24, 72, 168, and 240 h, fresh medium was substituted for the PBS (pH 7.4 and 5.5) release medium. Data were evaluated using the Peppas model of SigmaPlot v. 10.0 (Systat Software, Inc., San Jose, CA, USA).

### 2.11. In Vivo Pharmacokinetic Study

Sprague–Dawley rats (male, 7–8 weeks old) were purchased from Samtako Bio Korea (Osan, Korea) for pharmacokinetic and biodistribution studies. The animal experiments in this study were approved by the Institutional Animal Care and Use Committee (IACUC) of Chungbuk National University (No. CBNUA-1711-22-01, 27 April 2022). All rats used in the experiment were housed in a constant temperature–humidity and well-ventilated environment and were provided with sufficient food and water. The rats were administered either PTX/ETP/RAPA-loaded mPEG-pH-PCL micelles or PTX/ETP/RAPA solution through the tail vein. The PTX/ETP/RAPA solution was dissolved in EtOH:Cremophor EL^®^ (50:50, *v/v*) and was used as a control. The respective doses of PTX, ETP, and RAPA were 10 mg/kg, 10 mg/kg, and 5 mg/kg. At 5, 15, 30, 60, 120, 240, and 480 min after administration, blood (<500 μL) was collected from the retro-orbital plexus and centrifuged at 5000 rpm for 5 min to obtain plasma samples. Plasma samples were stored in a −70 °C deep freezer and were analyzed according to the pretreatment procedure described in the Section 2.13.2. The pharmacokinetic parameters of PTX, ETP, and RAPA were analyzed using a one-compartment model and were calculated using SigmaPlot v. 10.0 (Systat Software, Inc., San Jose, CA, USA).

### 2.12. Biodistribution Study

A biodistribution study was conducted to assess the tissue distribution of the drug at 8 h after intravenous injection of PTX/ETP/RAPA-loaded mPEG-pH-PCL micelles and PTX/ETP/RAPA solution. The rats were euthanized with CO_2_ gas, and liver, spleen, kidney, heart, lung, and muscle tissues were collected. Tissue samples were cleaned with PBS and stored in a −70 °C deep freezer until analysis.

### 2.13. HPLC Analysis

#### 2.13.1. Assay Conditions

The HPLC system (Waters, Milford, MA, USA), consisting of a 2996 photodiode array detector and a 2695 separation module, was used to detect the concentration of PTX, ETP, RAPA, and sorafenib (internal standard, IS). For HPLC analysis, a Fortis C18 HPLC column (5 µm, 4.6 × 250 mm) (Fortis^®^ Technologies Ltd., Cheshire, UK) was used, and the column temperature was maintained at 30 °C. The sample injection volume was 10 µL, and the isocratic mode was used to elute PTX, ETP, RAPA, and IS. The ACN:DW ratio of the mobile phase was 70:30 (*v*/*v*), and the flow rate was 1.0 mL/min. The retention times of PTX, ETP, RAPA, and IS were 5.8, 2.8, 21.7, and 8.9 min, and the wavelengths were detected at 227, 284, 277, and 264 nm, respectively. Each drug concentration was determined by substituting its peak area into a standard curve.

#### 2.13.2. Preparation of Biological Samples

Frozen plasma samples were thawed at 25 ℃ room temperature, and 50 μL of IS and 400 μL of MeOH were added to 200 μL of plasma sample. The mixture was vortexed and centrifuged for 5 min at 13,000 rpm. The supernatant was passed through a 0.2 μm sterile filter, and the concentrations of PTX, ETP, RAPA, and IS were measured by HPLC. The amounts of PTX, ETP, and RAPA in each tissue were measured using the homogenization method [54]. Briefly, tissue samples were homogenized in an Ultra-Turrax T25 homogenizer (IKA Works Inc., Staufen, Germany) using a Teflon pestle. Then, 200 μL of tissue sample was pretreated in the same manner as above, and the concentration of each drug was analyzed by HPLC.

### 2.14. Statistical Analysis

All data are presented as the mean ± standard deviation (SD). Drug release experiment results are expressed as the mean ± standard error (SE). All experiments were performed more than three times. An unpaired *t*-test was conducted for the statistical analysis with GraphPad Prism v. 5.0 (GraphPad Software, La Jolla, CA, USA); a *p*-value < 0.05 was regarded as statistically significant.

## 3. Results

### 3.1. Evaluation of the Synergistic Effect of PTX, ETP, and RAPA

Table 1 shows the IC_50_ values of PTX, ETP, and RAPA in two ratios and the CI values of each ratio. PTX and ETP exhibited similar IC_50_ values in both ratios, while RAPA showed a lower IC_50_ value in the 2:2:1 ratio, showing a significant difference (*p* < 0.05). As a result of CI value analysis, the 2:2:1 and 1:1:1 ratios both showed a synergistic effect with a CI value of 0.06.

### 3.2. Physicochemical Characterization of PTX/ETP/RAPA-Loaded mPEG-pH-PCL Micelles

**Figure 2:**
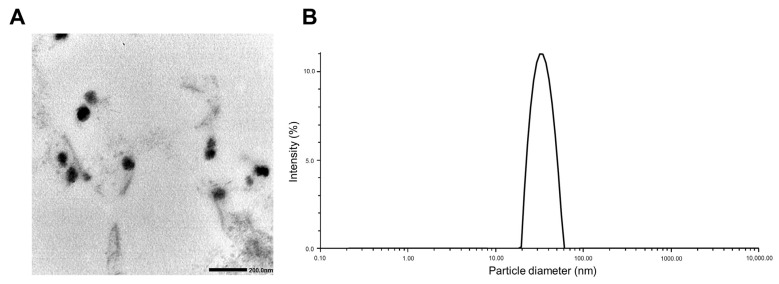
(**A**) Transmission electron microscopy (TEM) image of PTX/ETP/RAPA-loaded mPEG-pH-PCL micelles. (**B**) Representative particle size distributions of PTX/ETP/RAPA-loaded mPEG-pH-PCL micelles (n = 3).

Table 2 shows the EE (%), DL (%), particle size, zeta potential, and PDI of PTX/ETP/RAPA-loaded mPEG-pH-PCL micelles at a 2:2:1 ratio. The EE (%) of PTX, ETP, and RAPA encapsulated in mPEG-pH-PCL micelles were 64.8%, 67.3%, and 70.3%, and the DL (%) was 2.49%, 2.59%, and 1.38%, respectively. In addition, the particle size was 35.0 ± 0.24 nm, the PDI value was 0.03 ± 0.70, and the zeta potential was −0.22 ± 0.03 mV. The morphology and dispersion of the PTX/ETP/RAPA-loaded mPEG-pH-PCL micelles were characterized by TEM and DLS. The TEM image indicates that the morphology of the micelles is uniformly spherical (Figure 2A), and the particle size distribution indicates that the micelle size is <100 nm (Figure 2B).

### 3.3. In Vitro pH-Sensitivity Assessment of PTX/ETP/RAPA-Loaded mPEG-pH-PCL Micelles

Table 3 shows the particle size, zeta potential, and PDI of PTX/ETP/RAPA-loaded mPEG-pH-PCL micelles at a pH of 7.4 and a pH of 5.5. At a pH of 7.4, the size of the micelles increased significantly at 8 h compared to 0 h (Table 3A). On the other hand, at a pH of 5.5, the size of the micelles increased significantly at 6 h compared to 0 h, which is 2 h faster than at a pH of 7.4 (Table 3B) (*p* < 0.05).

### 3.4. In Vitro Cytotoxicity Assay

**Figure 3:**
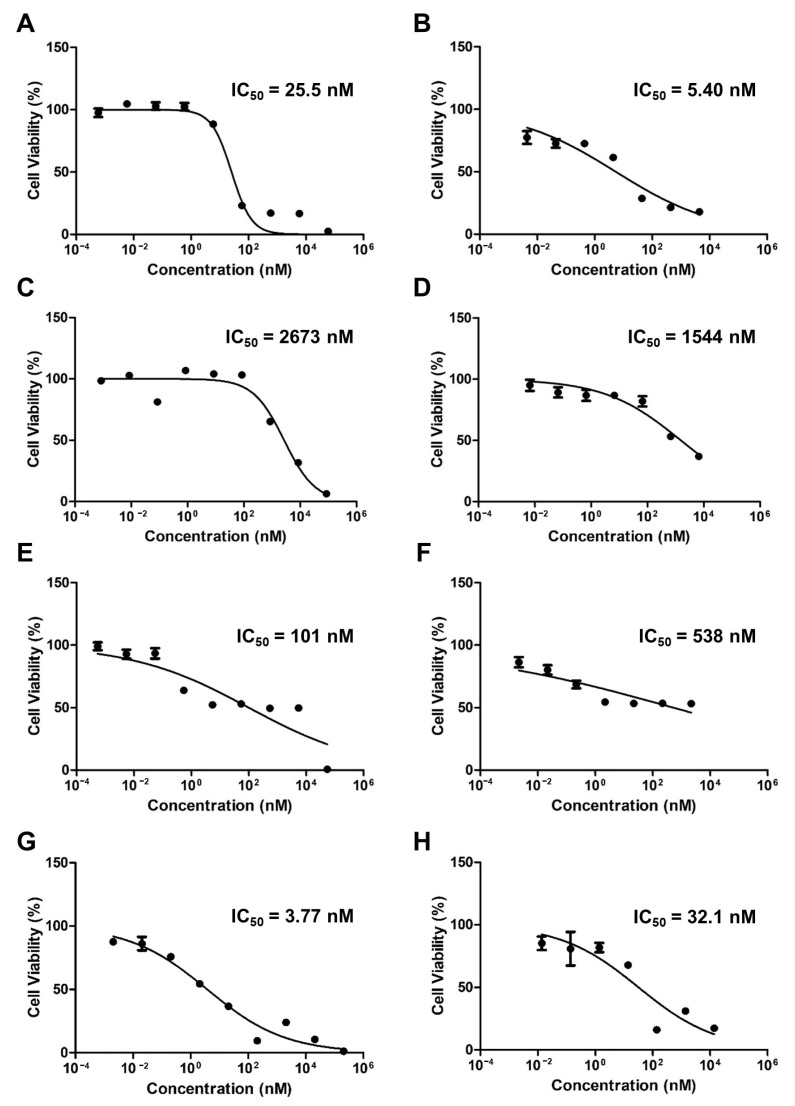
In vitro cytotoxicity analysis of AGS-Luc2 cells after 48 h of treatment with (**A**) free PTX, (**B**) PTX-loaded mPEG-pH-PCL micelles, (**C**) free ETP, (**D**) ETP-loaded mPEG-pH-PCL micelles, (**E**) free RAPA, (**F**) RAPA-loaded mPEG-pH-PCL micelles, (**G**) free PTX/ETP/RAPA, and (**H**) PTX/ETP/RAPA-loaded mPEG-pH-PCL micelles (n = 18).

Figure 3 shows the cytotoxicity results for the free drugs and micelles when PTX, ETP, and RAPA were used alone or in combination. The IC_50_ value of free PTX was 25.5 nM and the PTX-loaded mPEG-pH-PCL micelle was 5.40 nM (Figure 3A,B). The IC_50_ value of free ETP was 2673 nM and the ETP-loaded mPEG-pH-PCL micelle was 1544 nM (Figure 3C,D). The IC_50_ value of free RAPA was 101 nM and the RAPA-loaded mPEG-pH-PCL micelle was 538 nM (Figure 3E,F). Finally, the IC_50_ value of free PTX/ETP/RAPA was 3.77 nM and the PTX/ETP/RAPA-loaded mPEG-pH-PCL micelle was 32.1 nM (Figure 3G,H).

### 3.5. In Vitro Clonogenic Assay

**Figure 4:**
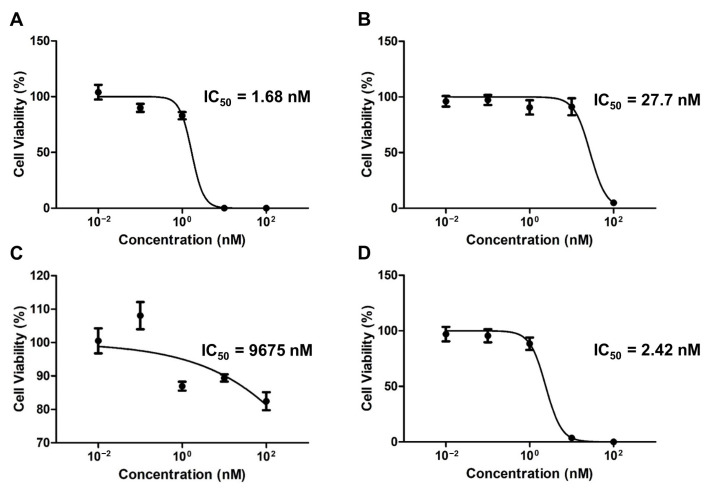
In vitro clonogenic analysis of AGS-Luc2 cells after two weeks of treatment with (**A**) PTX-loaded mPEG-pH-PCL micelles, (**B**) ETP-loaded mPEG-pH-PCL micelles, (**C**) RAPA-loaded mPEG-pH-PCL micelles, and (**D**) PTX/ETP/RAPA-loaded mPEG-pH-PCL micelles (n = 3).

The colony inhibition rate of the drug-encapsulated micelles was evaluated using a clonogenic assay over two weeks (Figure 4). The respective IC_50_ values of the PTX-, ETP-, RAPA-, and PTX/ETP/RAPA-loaded mPEG-pH-PCL micelles were 1.68 nM (Figure 4A), 27.7 nM (Figure 4B), 9675 nM (Figure 4C), and 2.42 nM (Figure 4D). The CI value was 0.54, indicating synergism.

### 3.6. In Vitro Drug Release Assay

**Figure 5:**
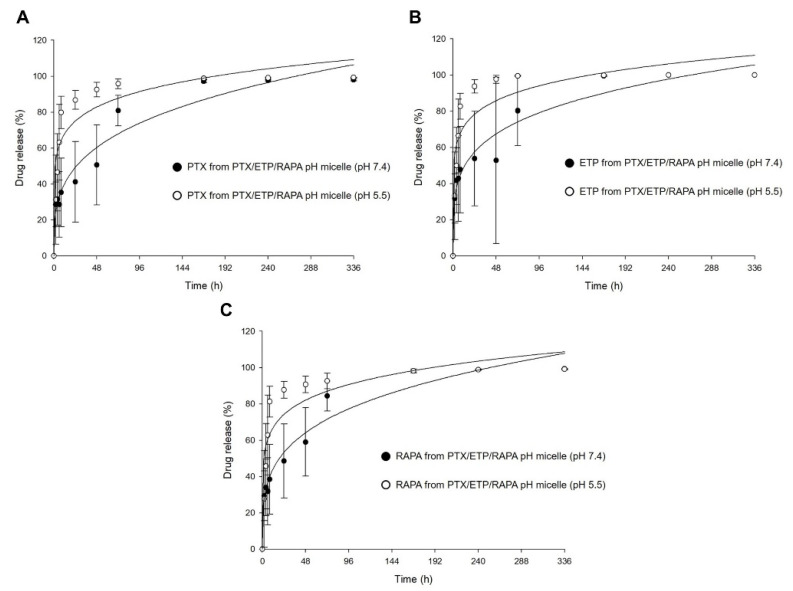
In vitro release profiles at different pH values of (**A**) PTX, (**B**) ETP, and (**C**) RAPA release in PTX/ETP/RAPA-loaded mPEG-pH-PCL micelles (n = 3–4).

Figure 5 shows the in vitro release profiles of PTX, ETP, and RAPA in PTX/ETP/RAPA-loaded mPEG-pH-PCL micelles. In Figure 5A, the 48 h release rate of PTX was 50% at a pH of 7.4 and 92% at a pH of 5.5. The 48 h release rate of ETP was 52% at a pH of 7.4 and 97% at a pH of 5.5 (Figure 5B). The 48 h release rate of RAPA was 59% at a pH of 7.4 and 90% at a pH of 5.5 (Figure 5C). At a pH of 5.5, PTX showed a rapid release rate >90% after 48 h, ETP after 24 h, and RAPA after 48 h. In contrast, at a pH of 7.4, all three drugs showed a release rate >90% after 168 h. Consequently, the drug release for the PTX/ETP/RAPA-loaded mPEG-pH-PCL micelles at a pH of 5.5 was much faster than at a pH of 7.4.

### 3.7. In Vivo Pharmacokinetic Study

**Figure 6:**
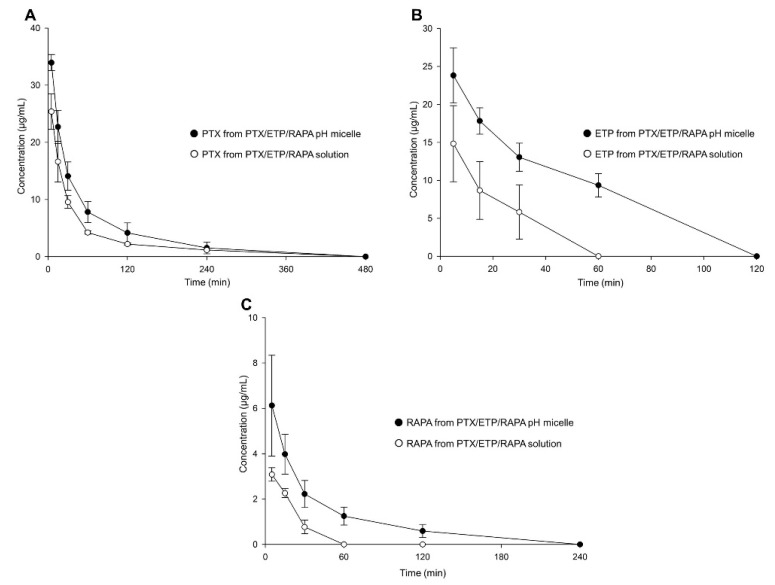
Plasma concentration–time profiles of PTX, ETP, and RAPA after intravenous injection. (**A**) PTX, (**B**) ETP, and (**C**) RAPA concentration in PTX/ETP/RAPA-loaded mPEG-pH-PCL micelles and PTX/ETP/RAPA solution (n = 3).

Figure 6 and Table 4 show the plasma concentration–time profiles and pharmacokinetic parameters of the PTX/ETP/RAPA-loaded mPEG-pH-PCL micelles and the PTX/ETP/RAPA solution. In Figure 6A, the plasma concentrations of PTX were detected at up to 240 min, both in the micelles and in the solution. Those of ETP were detected at up to 60 min in the micelles and at up to 30 min in the solution (Figure 6B). Finally, the plasma concentrations of RAPA were detected at up to 120 min in the micelles and at up to 30 min in the solution (Figure 6C). All three drugs were not found at concentrations below the limit of detection (LOD). In Table 4, the PTX area-under-the-curve (AUC) value of the PTX/ETP/RAPA-loaded mPEG-pH-PCL micelles was 1.6-fold higher in the micelles than in the solution. The AUC value of ETP was 3.0-fold higher in the micelles than in the solution (*p* < 0.05), and that of RAPA was 2.4-fold higher in the micelles than in the solution (*p* < 0.05). Overall, the micelle formulation showed higher AUC values and lower total clearance (CL_t_) and volume of distribution (V_d_) values than the solution.

### 3.8. Biodistribution Study

**Figure 7:**
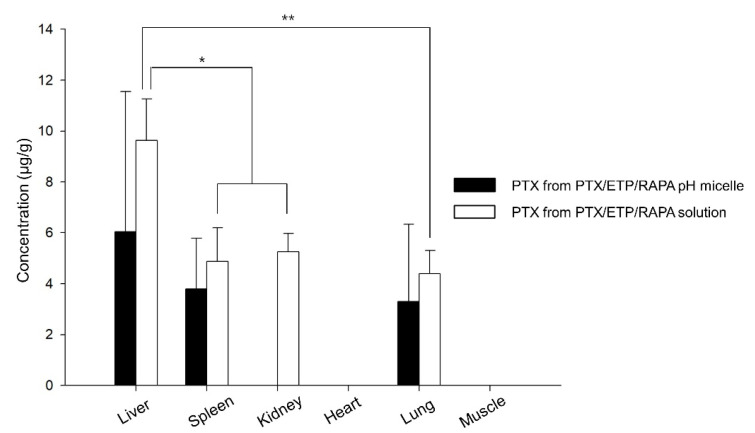
Biodistribution of PTX in PTX/ETP/RAPA-loaded mPEG-pH-PCL micelles and PTX/ETP/RAPA solution in each tissue at 8 h after intravenous injection (* *p* < 0.05, ** *p* < 0.01) (n = 3).

Figure 7 shows the drug distribution of the PTX/ETP/RAPA-loaded mPEG-pH-PCL micelles and the PTX/ETP/RAPA solution in major organs 8 h after intravenous injection. PTX was detected in four organs, whereas ETP and RAPA were not detected in the liver, spleen, kidneys, heart, lungs, and muscles. The solution was detected in the liver, spleen, kidneys, and lungs, but micelles were not detected in the kidneys due to values below the LOD. The highest amount of PTX was detected in the liver for both the micelles and solutions, and the solution was detected at a 1.6-fold higher concentration than that of the micelles. Overall, a higher concentration of PTX was detected in the solution than in the micelles.

## 4. Discussion

PTX, ETP, and RAPA have been reported to be effective against various cancers, and due to the different mechanisms of each drug, they can simultaneously target multiple pathways of cancer cells when combined [55,56]. However, all three drugs are limited in their clinical application due to their low solubility in water [57,58,59]. Therefore, pH-sensitive polymeric micelles were used to improve the water solubility of PTX, ETP, and RAPA, and effectively control the drug release from cancer cells. These pH-sensitive polymeric micelles have attracted much attention as an effective strategy for the targeted delivery of antitumor drugs. Based on previous studies, pH-sensitive polymeric micelles are prepared using the mPEG-pH-PCL polymer [43]. As a result, we expected to observe synergism of the three drugs in GC and the controlled drug release of the micelles.

To evaluate the synergistic effect of PTX, ETP, and RAPA in GC, CI values were evaluated at the weight ratios of 2:2:1 and 1:1:1. Results showed that both ratios had a CI value of 0.06, indicating synergism. Intravenous doses of PTX, ETP, and RAPA have been reported to be 10–20 mg/kg, 10–30 mg/kg, and 2–10 mg/kg, respectively [60,61,62,63,64,65,66,67]. Consequently, we chose a final ratio of 2:2:1, considering that RAPA has a relatively small intravenous dose and that RAPA exhibited a lower IC_50_ value at the 2:2:1 ratio. The evaluation of the physicochemical properties by manufacturing micelles at a ratio of 2:2:1 revealed that the EE (%) of all three drugs was >60% and that the spherical micelles had a particle size of <100 nm. Such small particle sizes (< 100 nm) enhanced the vascular permeability at the target site, favoring preferential accumulation at the tumor site [68]. Moreover, the size of the micelle increased at a faster rate at a pH of 5.5 than at a pH of 7.4. These results indicate that citraconic amide bonds are cleaved and micelles are dissociated under weakly acidic conditions, resulting in an enlarged hydrodynamic diameter.

The IC_50_ values of free PTX/ETP/RAPA were lower than those of the PTX/ETP/RAPA-loaded mPEG-pH-PCL micelles according to in vitro cytotoxicity analyses. These results may be related to the in vitro drug release profiles. In the PTX/ETP/RAPA-loaded mPEG-pH-PCL micelles, the 48 h release rates of PTX, ETP, and RAPA were 50%, 52%, and 59%, respectively. Therefore, considering the slow release rate of the drug-encapsulated in micelles at 48 h and the fact that endocytosis of micelles may require time, micelles are considered to have a lower cytotoxicity than free drugs [69,70]. As a result of evaluating the long-term colony suppression rate for two weeks in the in vitro clonogenic assay, the CI value of the PTX/ETP/RAPA-loaded mPEG-pH-PCL micelles was shown to be 0.54, indicating synergism. The in vitro drug release profiles showed that the release rate of each drug increased as the pH value decreased for the PTX/ETP/RAPA-loaded mPEG-pH-PCL micelles. These results indicate that the release of the drug is accelerated by breaking the citraconic amide bond at a slightly acidic pH of 5.5. Therefore, in vitro release results suggest that PTX/ETP/RAPA-loaded mPEG-pH-PCL micelles are pH-dependent and exhibit different drug release behaviors depending on the pH of the tumors and normal tissues, which will be effective in treating targeted cancer.

In vivo pharmacokinetic studies have shown that PTX/ETP/RAPA-loaded mPEG-pH-PCL micelles are detected at higher concentrations in the blood for a longer time than solutions. The AUC values of PTX, ETP, and RAPA in the micelles were 1.6-, 3.0-, and 2.4-fold higher than in their respective solutions. In addition, the clearance values were 1.5-, 3.8-, and 2.3-fold lower in the micelles than in their solutions, respectively. These results suggest that micelle formulations can promote long-lasting drug release in the body through improved bioavailability and increased circulation time in the blood than solutions [71]. In the biodistribution study, PTX was detected most in the liver, both in the micelles and in the solution, which is interpreted as drug accumulation due to absorption of the reticuloendothelial system (RES) [72]. Moreover, PTX accumulation in the micelles was lower than in the solution, indicating that the micelle formulation could reduce hepatotoxicity [73]. Regarding the PK parameter table, the solution accumulates more in the organs because the solution had higher CL_t_ and V_d_ values than the micelles [74].

## 5. Conclusions

In conclusion, we evaluated the synergistic effects of PTX, ETP, and RAPA and evaluated the physicochemical properties of PTX/ETP/RAPA-loaded mPEG-pH-PCL micelles at a 2:2:1 ratio. In vitro studies showed that the size of micelles increases more rapidly at a pH of 5.5 than at a pH of 7.4, and the release rate of each drug increases with decreasing pH values in PTX/ETP/RAPA-loaded mPEG-pH-PCL micelles. In vivo studies demonstrated that micelle formulations exhibit an improved bioavailability and an increased circulation time in the blood than solutions. Therefore, we suggest that PTX/ETP/RAPA-loaded mPEG-pH-PCL micelles are an advantageous drug delivery strategy for GC treatment.

## Figures and Tables

**Table 1:** Half-maximal inhibitory concentration (IC_50_) and combination index (CI) values at two ratios of paclitaxel (PTX), etoposide (ETP), and rapamycin (RAPA) (n = 18).

PTX:ETP:RAPA (Weight Ratio)	IC_50_ (nM)			CI Value
	PTX	ETP	RAPA	
2:2:1	1.32 ± 0.44	1.92 ± 0.63	0.62 ± 0.20	0.06 ± 0.02
1:1:1	1.20 ± 0.09	1.73 ± 0.13	1.12 ± 0.09	0.06 ± 0.00

**Table 2:** Physicochemical characterization of paclitaxel (PTX), etoposide (ETP), and rapamycin (RAPA)-loaded mPEG-pH-PCL micelles (n = 3).

Formulation	Amount of Polymer Used (mg)	Encapsulation Efficiency (EE %)			Drug Loading (DL %)			Particle Size (nm)	Poly-Dispersity Index (PDI)	Zeta Potential (mV)
		PTX	ETP	RAPA	PTX	ETP	RAPA			
PTX:ETP:RAPA (2:2:1)	150	64.8 ± 1.85	67.3 ± 1.41	70.3 ± 2.71	2.49 ± 0.07	2.59 ± 0.05	1.38 ± 0.05	35.0 ± 0.24	0.03 ± 0.70	−0.22 ± 0.03

**Table 3:** Particle size, zeta potential, and poly-dispersity index (PDI) of paclitaxel (PTX), etoposide (ETP), and rapamycin (RAPA)-loaded mPEG-pH-PCL micelles at (**A**) a pH of 7.4 and (**B**) a pH of 5.5 (n = 3).

**(A)**					
**Time (h)**	**0**	**2**	**4**	**6**	**8**
Particle size (nm)	35.0 ± 0.24	41.0 ± 0.71	48.7 ± 3.04	66.9 ± 23.4	433,501 ± 612,891
PDI	0.03 ± 0.70	0.22 ± 0.46	0.28 ± 1.15	0.26 ± 4.58	1.92 ± 247
Zeta potential (mV)	−0.22 ± 0.03	−2.90 ± 0.70	−0.33 ± 0.20	−0.13 ± 0.03	−5.22 ± 8.26
**(B)**					
**Time (h)**	**0**	**2**	**4**	**6**	**8**
Particle size (nm)	35.1 ± 0.31	41.4 ± 2.30	50.4 ± 3.36	38,5002 ± 168,486	118,618 ± 166,924
PDI	0.04 ± 1.11	0.23 ± 1.48	0.26 ± 2.16	1.07 ± 111	0.75 ± 69.6
Zeta potential (mV)	−0.08 ± 0.18	−1.30 ± 0.58	0.55 ± 0.85	−0.17 ± 0.15	−0.03 ± 0.08

**Table 4:** Pharmacokinetic parameters of paclitaxel (PTX), etoposide (ETP), and rapamycin (RAPA) in PTX/ETP/RAPA-loaded mPEG-pH-PCL micelles and PTX/ETP/RAPA solution after intravenous injection (n = 3).

Parameters	PTX in Combination Solution	PTX in Combination Micelle	ETP in Combination Solution	ETP in Combination Micelle	RAPA in Combination Solution	RAPA in Combination Micelle
Dose (µg∙kg^−1^)	10,000	10,000	10,000	10,000	5000	5000
AUC (min∙µg∙mL^−1^)	821 ± 127	1300 ± 314	445 ± 231	1331 ± 221	87.4 ± 15.4	206 ± 40.5
CL_t_ (mL∙kg^−1^∙min)	12.4 ± 1.98	8.01 ± 1.98	29.1 ± 19.8	7.66 ± 1.34	58.3 ± 9.37	24.9 ± 5.31
V_d_ (mL∙kg^−1^)	340 ± 52.1	264 ± 17.3	579 ± 147	403 ± 56.3	1245 ± 145	783 ± 327
t_1/2_ (min)	19.1 ± 1.70	23.9 ± 7.05	16.3 ± 5.62	37.1 ± 7.00	15.1 ± 3.39	21.4 ± 6.02

Abbreviations: AUC, area under the curve; CL_t_, total clearance; V_d_, volume of distribution.

## Data Availability

The data presented in this article are contained in the manuscript.
